# Loss of SLC9A3 decreases CFTR protein and causes obstructed azoospermia in mice

**DOI:** 10.1371/journal.pgen.1006715

**Published:** 2017-04-06

**Authors:** Ya-Yun Wang, Ying-Hung Lin, Yi-No Wu, Yen-Lin Chen, Yung-Chih Lin, Chiao-Yin Cheng, Han-Sun Chiang

**Affiliations:** 1 Department of Chemistry, Fu Jen Catholic University, New Taipei City, Taiwan; 2 Graduate Institute of Biomedical and Pharmaceutical Science, Fu Jen Catholic University, New Taipei City, Taiwan; 3 Department of Pathology, Cardinal Tien Hospital, New Taipei City, Taiwan; 4 Department of Urology, Taipei Medical University Hospital, Taipei, Taiwan; University of Nevada School of Medicine, UNITED STATES

## Abstract

Mutations in the cystic fibrosis transmembrane conductance regulator (*CFTR*) gene cause cystic fibrosis (CF) and are associated with congenital bilateral absence of the vas deferens (CBAVD), which is the major cause of infertility in male patients with CF. However, most Taiwanese patients with CBAVD do not carry major *CFTR* mutations. Some patients have a single copy deletion of the solute carrier family 9 isoform 3 (*SLC9A3*) gene. SLC9A3 is a Na^+^/H^+^ exchanger, and depleted *Slc9a3* in male mice causes infertility due to the abnormal dilated lumen of the rete testis and efferent ductules. Furthermore, SLC9A3 interacts with CFTR in the pancreatic duct and functions as a genetic modifier of CF. However, SLC9A3 function and its relation to CFTR expression in the male reproductive tract in vivo remain elusive. In the present study, we found that CFTR expression was dramatically decreased in the epididymis and vas deferens of *Slc9a3* knockout mice. Adult *Slc9a3*^*-/-*^ mice showed not only significantly decreased epididymis and vas deferens weight but also increased testis weight. Furthermore, *Slc9a3*^*-/-*^ mice developed obstructive azoospermia because of abnormal abundant secretions and calcification in the lumen of the reproductive tract. Ultrastructural analysis of the epithelium in *Slc9a3*^*–/–*^epididymis and vas deferens displayed disorganized and reduced number of stereocilia and numerous secretory apparatuses. Our data revealed that interdependence between SLC9A3 and CFTR is critical for maintaining a precise microenvironment in the epithelial cytoarchitecture of the male reproductive tract. The *Slc9a3*-deficient mice with impaired male excurrent ducts in this study provide proof for our clinical findings that some Taiwanese of CBAVD carry *SLC9A3* deletion but without major *CFTR* mutations.

## Author summary

Cystic fibrosis (CF) is the most common inherited life-threatening disease in Caucasians. The most well-known cause of CF is a genetic defect in *CFTR*, an apical membrane chloride and bicarbonate channel. The symptoms of CF include defects in the respiratory, digestive, and male reproductive systems. Most male patients with CF are infertile due to congenital bilateral absence of the vas deferens (CBAVD), which leads to obstructive azoospermia. Nevertheless, Taiwanese patients with CBAVD do not carry the common mutations of *CFTR* found in Caucasians. We have identified a potential candidate, *SLC9A3*, of which a single copy is lost in Taiwanese patients with CBAVD. In addition to the previously reported role of *SLC9A3* in the digestive system and efferent ductules, we now report that the SLC9A3 deficiency causes obstructive azoospermia and impairs the epithelial structure of the reproductive tract. Loss of SLC9A3 also leads to dramatic reduced expression of CFTR in the reproductive tract. We suggest that the interplay between SLC9A3 and CFTR is responsible for CF-related infertility. Thus, we have characterized a potential critical player in the pathogenesis of CBAVD and provide a new diagnostic candidate for Asian patients with CBAVD.

## Introduction

### Pathology of cystic fibrosis and congenital bilateral absence of the vas deferens

Cystic fibrosis (CF), characterized by mutations in transmembrane conductance regulator (*CFTR*) gene, is the most common autosomal recessive disorder in Caucasians [1–4]. CFTR is an apical membrane Cl^-^ channel and is responsible for anion secretion in the lungs, pancreas, male reproductive tract, and other epithelial cells. Loss of CFTR activity causes dehydration of the apical membrane and impairs the clearance of mucus from the respiratory tract [5]. Most patients with congenital bilateral absence of the vas deferens (CBAVD) have mutations in and/or susceptible variants of the 5T allele in intron 8 of *CFTR* [6–8]. Up to 78%–82% of genetic mutations or variants of *CFTR* have been detected in CBAVD patients from different countries [6,8–13]. Abnormal atrophy of the tissue structure of the vas deferens and the corpus and cauda epididymis is the major cause of male infertility in patients with CBAVD [14,15]. However, most Taiwanese patients with CBAVD do not carry *CFTR* mutations, and this is consistent with the low incidence of CF in Asian populations including Taiwan [16]. We previously performed genome-wide mapping of copy-number variations through oligonucleotide array-based comparative genomic hybridization (CGH) and identified loss of solute carrier family 9 isoform 3 (*SLC9A3*) allele in Taiwanese men with CBAVD (in two of seven Taiwanese men with CBAVD) [17].

### Functional roles of SLC9A3

SLC9A3, a Na^+^/H^+^ exchanger, is expressed in the apical membranes of epididymal, vas deferens, renal proximal tubule, and intestinal epithelium [18–22]. Zhou et al. indicated that SLC9A3 is also expressed in the nonciliated cells of the efferent duct, which connects the testis and epididymis, and that *Slc9a3*^*-/-*^ male mice are infertile because of the abnormal dilated lumen of the rete testis and efferent ductules [23]. However, the role of SLC9A3 in the epididymis and vas deferens remain to be clarified. Another well-known function of SLC9A3 is regulation of ion homeostasis in the intestine and colon. SLC9A3 is mainly involved in the transepithelial absorption of Na^+^ and water and often functionally couples with the Cl^-^/HCO3^-^ exchanger [24]. In one previous study, *Slc9a3*^*-/-*^ mice showed elevated intestinal fluid and diarrhea because of decreased Na^+^ and HCO_3_^-^ absorption [21].

### CFTR interacts with SLC9A3

Ahn et al. was the first to demonstrate that SLC9A3 interacts with the C-terminal PDZ motif of CFTR in PS120 cells [25]. In that study, SLC9A3 and CFTR were colocalised in the pancreatic duct of wild-type (WT) mice and SLC9A3 expression decreased by 53% in the pancreatic duct of homozygous △F508 mutation (*△F/△F*) *Cftr* mice. This reciprocal interaction between SLC9A3 and CFTR is regulated by sodium–hydrogen exchange regulatory cofactor 2 in a renal epithelial cell line [26]. Furthermore, loss of SLC9A3 activity increases survival and reduces the occurrence of intestinal obstructions in *Cftr*^*-/-*^ mice because it rescues the dehydration induced by impaired CFTR function in the intestinal epithelium [27].

### Clinical significance of single nucleotide polymorphisms in *SLC9A3* in CF

Genetic studies have also supported the clinical association between SLC9A3 and CF. Single nucleotide polymorphisms in *SLC9A3* in children with CF are significantly associated with two clinical manifestations, the early infection of *Pseudomonas aeruginosa* and worsened pulmonary function [28]. Genome-wide association studies have indicated that genetic variants of *SLC6A14*, *SLC26A9*, and *SLC9A3* in patients with CF (*n* = 3,763) increased susceptibility to early meconium ileus [29,30]. Furthermore, five CF-modifier loci, including *SLC9A3*, were associated with lung disease severity in 6,365 patients with CF [31]. These comprehensive studies highlight the critical associations between genetic variants of *SLC9A3* and clinical indices such as the penetrance of the phenotype and age of onset in patients with CF.

Although previous studies have indicated that SLC9A3 is associated with CFTR and significantly affects the severity of CF-related diseases, the direct connection between SLC9A3 and CF-related diseases in vivo is unclear. In the present study, we found that *Slc9a3* deficiency in mice induced CBAVD-like phenotypes.

## Results

### CFTR reduction may be responsible for the reproductive etiology of *Slc9a3*^*-/-*^ mice

**Fig 1:**
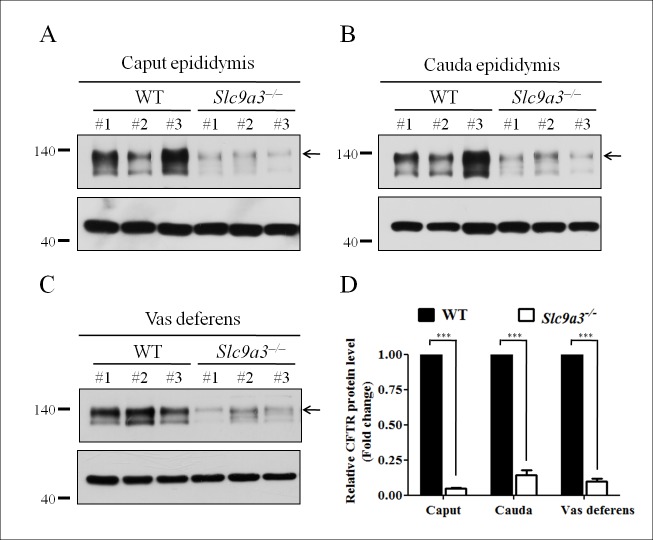
CFTR is prominently reduced in the epididymis and vas deferens of *Slc9a3*^-/-^ mice. (A, B, C) CFTR expression in the caput (A) and cauda (B) epididymis and vas deferens (C) of 2-month-old *Slc9a3*^*-/-*^ mice compared with that in WT mice was assessed by western blotting. In each panel, the upper image shows the CFTR expression (arrow indicates the fully glycosylated form) and the lower image shows the β-actin expression, which was used as a loading control. Each group comprised three WT and *Slc9a3*^*-/-*^ mice. (D) Western blotting results were quantified through a morphometric analysis. For each organ, the CFTR intensity was normalized to that of β-actin expression, and the mean intensity in WT mice was used as a reference to calculate the fold change in expression in *Slc9a3*^*-/-*^ mice. Each bar represents the mean ±SEM; *n* = 3 per genotype. *Significant difference compared with WT mice (***p < 0.0001, analyzed using the Student’s *t* test).

Most Caucasian patients with CBAVD show genetic mutations or variants of *CFTR* [6]. However, genes associated with CBAVD in Asian and Taiwanese populations are unclear [16,32]. Our previous large-scale genetic screening suggested that *SLC9A3* is a high-potential candidate gene for CBAVD [17]. SLC9A3 and CFTR are coexpressed in the pancreatic duct, and the amount of SLC9A3 was shown to be reduced in *△F/△F Cftr* mice [25]. In our results, *Slc9a3*^-/-^ mice were completely infertile compared with age-matched WT and heterozygous mice (Table 1). Therefore, we evaluated whether CFTR expression in *Slc9a3*^-/-^ mice was reduced and contributed to the sterility. In contrast to our expectations, CFTR expression was drastically reduced in the caput (95.2% ± 1.3) and cauda (85.7% ± 6.6) epididymis and vas deferens (90.4% ± 4.1) (Fig 1). These findings suggested that reduced CFTR expression was responsible for the reproductive tract pathology in *Slc9a3*^-/-^ mice.

**Table 1:** Comparison of fertile capability between wild-type, heterozygous, and homozygous *Slc9a3* knockout mice.

Mating genotype		The average number of pups per litter
Male	Female	
***Slc9a3***^***+/+***^ **× *Slc9a3***^***+/+***^		**9.80 ± 2.59**
***Slc9a3***^***+/–***^ **× *Slc9a3***^***+/+***^		**8.80 ± 1.92**
***Slc9a3***^***–/–***^ **× *Slc9a3***^***+/+***^		**0**^**a**^

The number of progeny from breeding *Slc9a3*^*+/+*^, *Slc9a3*^*+/–*^, and *Slc9a3*^*–/–*^male mice is presented as the mean ± SEM (*n* = 5 for each group).

a: Significantly different from WT and heterozygous mutant mice (P < 0.0001) according to an unpaired Student’s *t* test.

**Fig 2:**
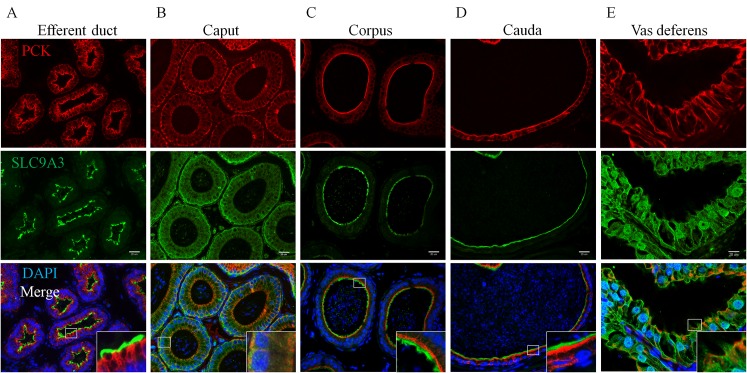
SLC9A3 expression in the epithelium of male excurrent ducts of 2-month-old WT mice. Localization of SLC9A3 (green, middle panel) and pan-cytokeratin (red, upper panel) in the efferent ducts (A); caput (B), corpus (C), and cauda (D) epididymis and vas deferens (E) of WT mice was detected by performing immunofluorescence double labelling with specific primary antibodies. Pan-cytokeratin was used as a specific marker of differentiated epithelial cells. The nucleus was stained with DAPI (blue) and is displayed in merged images (lower panel). The insets show higher magnification of the indicated boxed areas; scale bar = 20 μm.

Evaluating the causes of reduced CFTR expression and infertility in *Slc9a3*^-/-^ males first requires the precise localization of SLC9A3. Until now, information on the localization of SLC9A3 in different regions of the epididymis and vas deferens in mice has been incomplete. Several studies have shown that SLC9A3 is expressed in the apical region of nonciliated cells of the efferent ducts in species including humans, mice, rats, hamsters, and roosters [19,22,23,33–38]. Although the expression of SLC9A3 was detected in the principal cells of the epididymis except distal cauda epididymis and vas deferens in rats [22], additional details regarding SLC9A3 expression in mice remains to be revealed. We further verified the SLC9A3 localization in the epididymis and vas deferens of mice (Fig 2). The specificity of anti-SLC9A3 antibody used in our study was confirmed through immunofluorescence staining on the epididymal sections of two *Slc9a3*^*–/–*^mice (S1 Fig), and no signal was observed along the stereocilia in the epididymal epithelia of *Slc9a3*^-/-^ mice (S1A and S1B Fig). SLC9A3 localization in the efferent ducts was identical to that in previous studies (Fig 2A) [23,35]. In the epididymis of 2-month-old WT males, SLC9A3 was localized in the apical stereocilia of the corpus and cauda epididymal epithelia (Fig 2C and 2D). SLC9A3 signals were also detected along the stereocilia on the vas deferens epithelium (Fig 2E, enlarged box). By contrast, SLC9A3 was more widely distributed in the caput epididymal and vas deferens epithelia (Fig 2B and 2E). The SLC9A3 localization (Fig 2) corresponds to the significantly reduced expression of CFTR in *Slc9a3*-deficient epididymis and vas deferens (Fig 1). We speculate that the interaction between CFTR and SLC9A3 may be critical for protein stability in male excurrent ducts.

### Global effects in the reproductive system of *Slc9a3*^-/-^ mice

**Fig 3:**
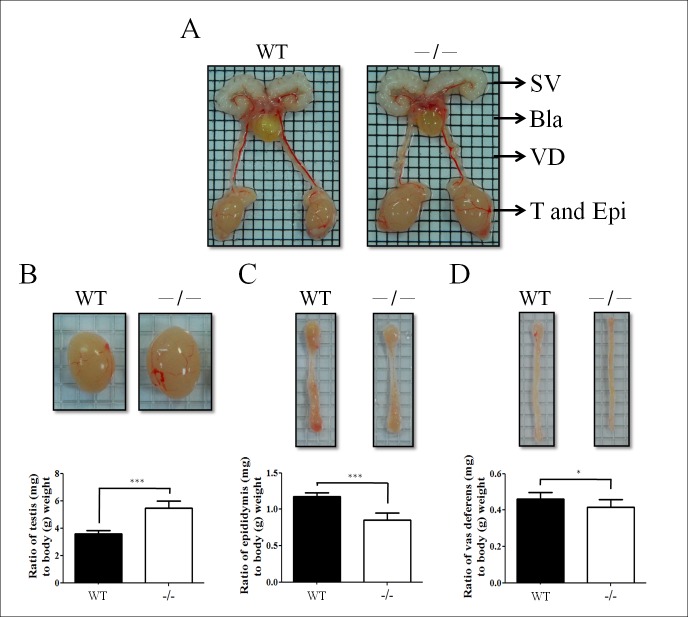
Gross morphology of the reproductive organs of infertile *Slc9a3*^*-/-*^ male mice. (A) Intact appearance of overall reproductive organs, including the seminal vesicle (SV), bladder (Bla), vas deferens (VD), testis (T), and epididymis (Epi), of 2-month-old WT and *Slc9a3* deficiency mice. (B, C, and D) Enlarged images of the testis (B), epididymis (C), and vas deferens (D). Statistics show the ratio of the normalized organ weight (mg) to body weight (g). Each bar represents the mean ± SEM; *n* = 10 per genotype. *Significant difference compared with WT mice (*p < 0.05 and ***p < 0.0001, analyzed using the Student’s *t* test).

Ten 2-month-old *Slc9a3*^-/-^ and WT male mice were analyzed to evaluate the pathology of the reproductive system. *Slc9a3*-deficient mice displayed increased testis size [3.59 ± 0.08 (WT) vs. 5.46 ± 0.17 (*Slc9a3*^*-/-*^)]. By contrast, the weight of the epididymis [1.18 ± 0.02 (WT) vs. 0.85 ± 0.02 (*Slc9a3*^*-/-*^)] and vas deferens [0.46 ± 0.01 (WT) vs. 0.41 ± 0.01 (*Slc9a3*^*-/-*^)] were reduced (Fig 3). We suggested that an obstruction probably occurred in the reproductive ducts of *Slc9a3*^-/-^ males and were similar to those observed in knockout (*cf/cf*) *Cftr* mice [39].

### Histopathological patterns of the testes and efferent ducts in *Slc9a3* knockout mice

**Fig 4:**
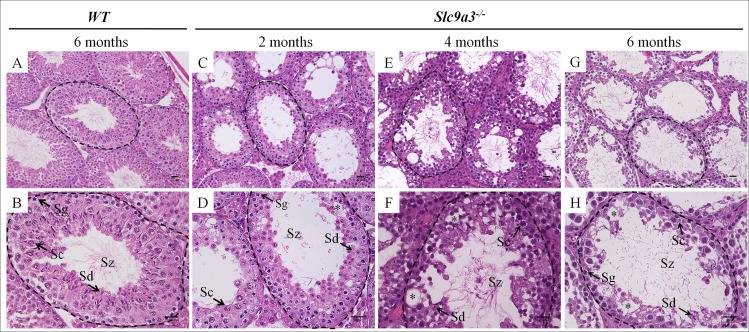
Loss of SLC9A3 induces testicular atrophy. Testicular histology was assessed by H&E staining of testis sections of 6-month-old WT mice (A, B) and *Slc9a3*^*-/-*^ mice of different ages (C–H). (A, B) Representative images showing the intact architecture of the seminiferous tubules and interstitial tissue in the testes of WT mice. (C–H) H&E staining of the testes of 2- (C, D), 4- (E, F), and 6–month-old (G, H) *Slc9a3*-deficient mice. Vacuolation of seminiferous epithelia was observed in the seminiferous tubules of *Slc9a3*^*-/-*^ mice (asterisk). The dashed line marks the intact lumen of the seminiferous tubules. The lower panel is the higher magnified images of the area indicated using a black dashed line in the upper panel; scale bar = 20 μm. Abbreviations: Sg, spermatogonia; Sc, spermatocyte; Sd, spermatid; Sz, spermatozoa.

To determine the effect of SLC9A3 deficiency on infertility, we first analyzed testicular sections of WT and *Slc9a3*^*-/-*^ mice of various ages to determine the course of progressive changes. The structures of the interstitial tissue and seminiferous tubules in WT testis were integrally organized, and germ cells showed regular arrangement and complete development (Fig 4A and 4B). The composition of the germ cell population was comparable between 2-month-old WT and *Slc9a3*^*-/-*^ mice, but the organization of germ cells in the *Slc9a3*^*-/-*^ mice was slightly disordered (Fig 4C and 4D). However, the number of each germ-cell type was slightly lower in 4 months-old *Slc9a3*^*-/-*^ males [Fig 4E and 4F; S2 Fig, the number of elongating spermatids: 144.25 ± 28.95 (WT) vs. 102.86 ± 26.9 (*Slc9a3*^*-/—*^ 4 months)]. Some testicular lumens of 6-month-old *Slc9a3*^*-/-*^ males had undergone atrophy and displayed moderate-to-severe hypospermatogenesis (Fig 4G and 4H).

**Fig 5:**
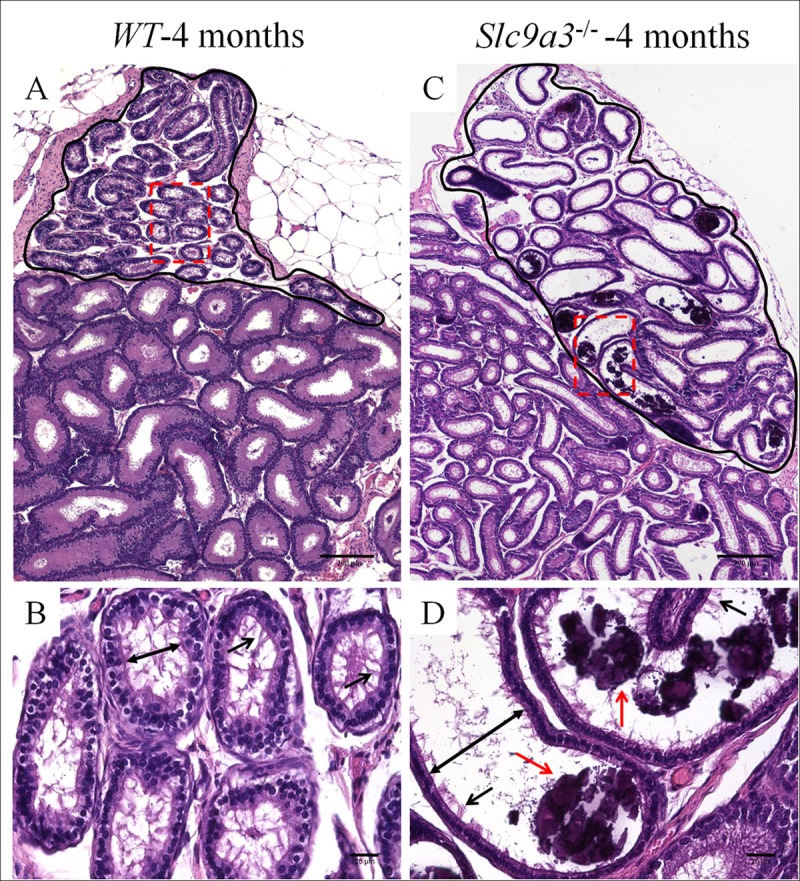
The lumen of the efferent ductules of 4-month-old *Slc9a3*^*-/-*^ males is dilated and calcified. Comparison of the efferent ducts of 4-month-old WT (A, B) and *Slc9a3*^*-/-*^ (C, D) mice according to H&E staining of the epididymal sections. (A, C) Each representation is a montage assembled by a sequence of film taken with a ×10 objective on the bright-field microscope. The circled region with black solid line in the upper part of the montage is the efferent ductule, and the lower part of the montage is the epididymis. (B, D) Enlarged images from the areas boxed by a red dashed box in the upper panel. The widest distance from one side to the other side of the lumen is marked by double-headed arrows, and cilia on the epithelium are indicated by black arrows. Calcification (red arrow) was observed in some dilated ducts. Scale bar = 200 μm (A, B) and 20 μm (C, D)

Efferent ductules, one of the factors underlying the *Slc9a3*^*-/-*^ testicular histopathology, are the connective bridge between the rete testis and epididymis and are involved in the reabsorption of approximately 90% of luminal fluid from the testes [40]. SLC9A3 was expressed on the epithelium of efferent ductules (Fig 2A). Compared with age-matched WT mice (Fig 5A and 5B), the luminal diameters of the efferent ductules in 4-month-old *Slc9a3*^*-/-*^ mice (Fig 5C and 5D) were wider. This phenotype was consistent with previous study by Zhou et al. [23]. Moreover, unexpected calcification was observed in the efferent ductules of *Slc9a3*^*-/-*^ mice at the age of 4 months (Fig 5C and 5D). According to these data, we speculated that the testes of *Slc9a3*^*-/-*^ mice underwent atrophy, probably because of back pressure from fluid accumulation caused by the obstruction and dysfunction of the efferent ducts [41–43].

### SLC9A3 depletion induces systemic defects in the excurrent ducts

**Fig 6:**
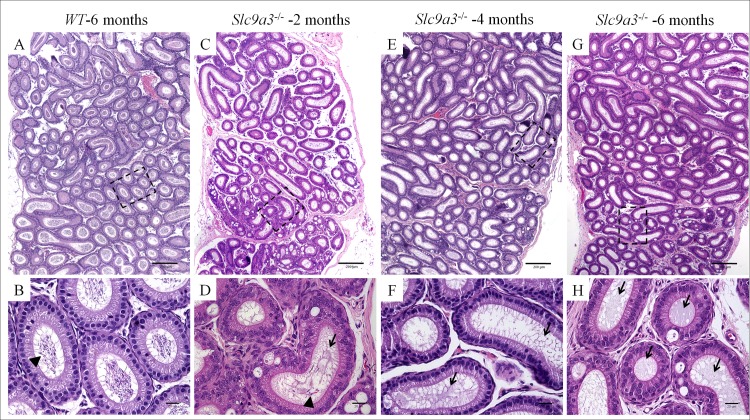
Absence of spermatozoa in the caput epididymis of adult *Slc9a3*^*-/-*^ mice. Histological pathology of the caput epididymis of 6-month-old WT (A, B) and 2- (C, D), 4- (E, F), and 6-month-old (G, H) *Slc9a3*^*-/-*^ mice was assessed by H&E staining. (A, C, E, G) Each montage, which contains a series of images obtained with a ×10 objective, displays the general morphology of the caput epididymis section. (B, D, F, H) Enlarged view is marked by a black dashed line in the upper panel. Spermatozoa are marked by arrowheads. Abnormal secretions (arrow) observed in the ducts of the caput epididymis of *Slc9a3*^*-/-*^ male mice. Scale bar = 200 μm (A, C, E, G) and 20 μm (B, D, F, H).

**Fig 7:**
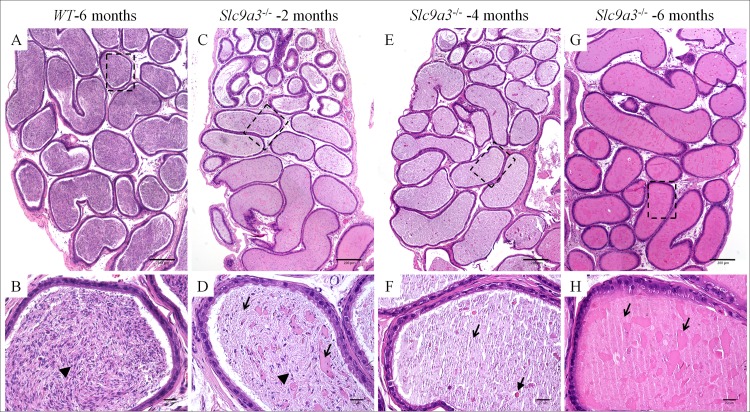
*Slc9a3*^*-/-*^ cauda epididymis is filled with abundant abnormal secretions instead of spermatozoa. H&E-stained sections of the cauda epididymis of 6 month-old WT (A, B) and *Slc9a3*^*–/–*^males aged 2 months (C, D), 4 months (E, F), and 6 months (G, H). (A, C, E, G) The low magnification overview is a montage assembled by an array of films continuously taken with a ×10 objective on a bright microscope. (B, D, F, H) Magnified view of the area indicated by a dashed box in the upper panel. Spermatozoa are indicated by arrowheads, and abnormal heterogeneous secretions are marked by arrows, a dashed arrow, and a green arrow. The white lines are sectioning artefacts. Scale bar = 200 μm (A, C, E, G) and 20 μm (B, D, F, H).

Sterility in patients with CF is due to the loss of the vas deferens and corpus and cauda epididymis [6]. We found that CFTR expression and organ weight were significantly decreased in the epididymis and vas deferens of *Slc9a3*^*-/-*^ mice (Figs 1 and 3). To characterize the physiological significance of SLC9A3 in the epididymis and vas deferens, we analyzed the histological changes in *Slc9a3*^*-/-*^ males of various ages. The lumen was filled with spermatozoa throughout the entire epididymis in WT mice (Fig 6A and 6B represent the caput epididymis; Fig 7A and 7B represent the cauda epididymis). In 2-month-old *Slc9a3*^*-/-*^ males, a reduced number of spermatozoa was observed in a few ducts of the caput epididymis (Fig 6C and 6D and S3 Fig). Recognizable spermatozoa were nearly absent in the lumen of the caput epididymis of >2-month-old *Slc9a3*^*-/-*^ males. Moreover, the level of abnormal secretions was augmented in the lumens of the caput epididymis with an increase in age of *Slc9a3*^*-/-*^ mice (Fig 6E–6H).

**Fig 8:**
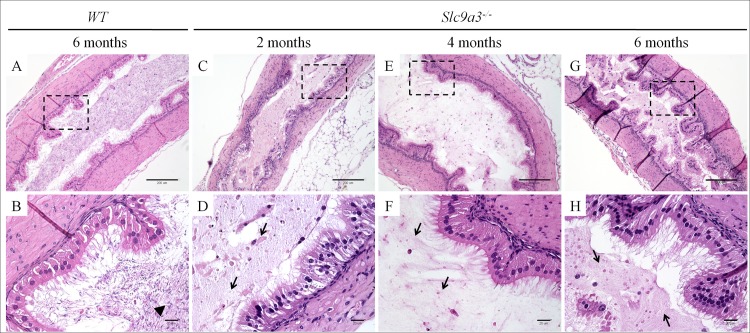
Aberrant secretions within the lumen of the vas deferens in *Slc9a3*^*-/-*^ males. Histology of the vas deferens of 6-month-old WT (A, B) and 2- (C, D), 4- (E, F), and 6-month-old (G, H) was examined by H&E-stained longitudinal sections. (A, C, E, G) General morphology at low magnification. (B, D, F, H) Micrograph showing a magnified view of the boxed area indicated in the upper panel. The arrowhead indicates spermatozoa. The arrow indicates abnormal heterogeneous secretions. The wrinkles on sections are sectioning artefacts. Scale bar = 200 μm (A, C, E, G) and 20 μm (B, D, F, H).

Similar phenotypes were occurred in the cauda epididymis of *Slc9a3*^*-/-*^ mice and were also found to progressively deteriorate with age (Fig 7). Compared with the caput epididymis of *Slc9a3*^*-/-*^ mice, the elevated secretions were significantly more severe in the cauda epididymis. The vas deferens of *Slc9a3*^*-/-*^ males also displayed elevated secretions (Fig 8). Spermatozoa were not transported from the epididymis into the vas deferens in 2-month-old *Slc9a3*^*-/-*^ mice (S3E and S3F Fig). Aberrant secreted materials were observed in the entire vas deferens, beginning from the age of 2 month. (Fig 8 and S4 Fig).

### SLC9A3 is essential for the integrity of the cytoarchitecture of the excurrent duct epithelium

**Fig 9:**
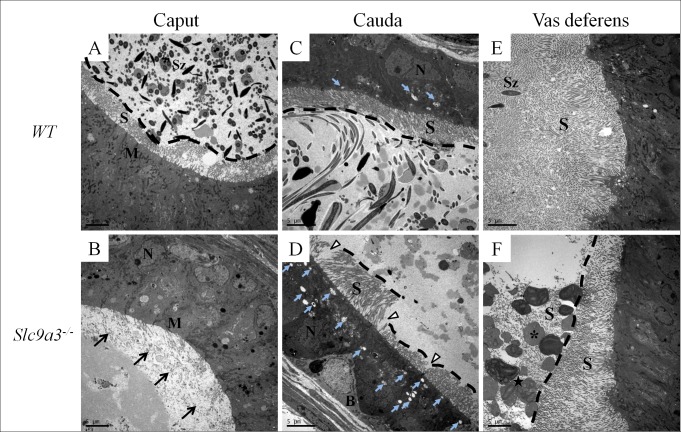
Ultrastructural defects of the epithelium in *Slc9a3*^*–/–*^epididymis and vas deferens. An electron micrograph (cross-section) showing the ultrastructure of the epithelia of the excurrent ducts of 2-month-old WT (upper panel) and *Slc9a3*^*-/-*^ male mice (lower panel). (A, B) Caput epididymis. The arrow indicates the absence of stereocilia on the caput epithelium. The black dashed line marks the boundary between the lumen and the edge of stereocilia on the epithelium. (C, D) Cauda epididymis. The white arrowhead indicates the disturbed and decreased number of stereocilia. The light blue arrow indicates the vesicles in the cauda epithelium. (E, F) Vas deferens. The asterisk indicates abnormal secretory particles and the star indicates smooth endoplasmic reticulum-like material in the particles (enlarged image in S2 Fig). The magnification for each image is slightly different, with 2000× magnification used for A, B, and E and 2500× magnification used for C, D, and F. Scale bar = 5 μm. Abbreviations: B, basal cell; M, mitochondria; N, nucleus; S, stereocilia; Sz, spermatozoa.

The epithelia of excurrent ducts are actively involved in the maintenance of proper luminal milieu through mechanisms including secretion and absorption [44]. Epididymal and vas deferens epithelium are lined by nonmotile stereocilia. These membrane-extended structures substantially increase the surface area and absorptive and secretive capacities of epithelial cells [44]. Because abundant secretions were existed in the excurrent ducts of *Slc9a3*^*-/-*^ mice, we further examined the ultrastructure of the epithelium in these organs by transmission electron microscopy. The amount of stereocilia was dramatically fewer in the *Slc9a3*^*-/-*^ caput epididymis (Fig 9A and 9B, arrows). Moreover, the stereocilia in the *Slc9a3*^*-/-*^ cauda epididymis was less organized and fewer in number. Vesicles, a type of secretory apparatus, were more abundant in the epithelium of the *Slc9a3*^*-/-*^ cauda epididymis (Fig 9C and 9D, light blue arrows). Distinct morphological changes were also observed in the vas deferens of *Slc9a3*^*-/-*^ mice. Stereocilia were less prominent and disturbed (Fig 9E and 9F). Hence, the less well-ordered and reduced number of stereocilia in the epithelium of the epididymis and vas deferens and more abundant secretory probably account for the chaotic secretion in the excurrent ducts of *Slc9a3*^*-/-*^ mice.

## Discussion

**Fig 10:**
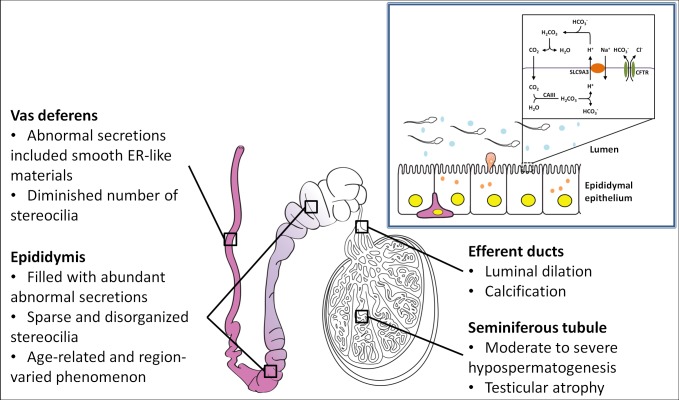
Proposed working model of the role of SLC9A3 in the pathogenesis of the male reproductive system. The upper right diagram depicts the possible cooperation between SLC9A3 and CFTR in regulating luminal homeostasis. The lower schematic diagram summarizes the pathological features of *Slc9a3*^*-/-*^ male mice. The color gradient in the epididymis represents the amount of abnormal heterogeneous secretions progressively increasing from the caput (light purple) to the cauda (dense purple) epididymis.

*SLC9A3*, one of CFTR interactor and genetic modifier of CF, is a candidate gene for CBAVD in Taiwanese patients. Our data indicated reduced CFTR levels and obstructed azoospermia-like phenotypes in the reproductive ducts of *Slc9a3*^*-/-*^ mice. Furthermore, the augmented secretions in the lumen are progressively worse through the epididymis to the vas deferens with an increase in the age. *Slc9a3*^*-/-*^ males also showed testicular atrophy, calcification in the efferent ducts, and impaired cytoarchitectures of the epithelium of the excurrent ducts. These results indicated a direct association between SLC9A3 and CBAVD in vivo (Fig 10).

### Progressive changes in the phenotypes of the efferent ductules of *Slc9a3*^*-/-*^ mice of different ages

Zhou et al. indicated that the luminal diameter of the efferent ductules in 90–120-day-old *Slc9a3*^*-/-*^ mice was dilated [23]. In our results, we further identified unexpected calcification in the lumen of the efferent ductules in *Slc9a3*^*-/-*^ mice at the age of 4 months. The expression of SLC9A3 is abundant in the apical membrane of the epithelium in the gastrointestinal tract and kidneys [45]. Pan et al. indicate that SLC9A3 plays a critical role not only in Na absorption but also in Ca^2+^ homeostasis [46]. In that study, *Slc9a3*^*-/-*^ mice exhibited reduced Ca^2+^ reabsorption in proximal tubules. SLC9A3-deficient mice also displayed lower intestinal Ca^2+^ absorption. Both of these contributed to the hypomineralized bones in *Slc9a3*^*-/-*^ mice. We suggest that SLC9A3 might participate in the regulation of Ca^2+^ homeostasis in the efferent ductules. SLC9A3 deficiency may have led to an imbalanced Ca^2+^ concentration and further depositing in the lumen of the efferent ductules as the *Slc9a3*^*-/-*^ mice aged. Because the efferent ductules are the bridge between the testis and epididymis in mice and they participate in the reabsorption of approximately 90% of luminal fluid from the testes, this obstruction in the efferent ductules caused the fluid accumulation and formation of back pressure to the testis of *Slc9a3*^*-/-*^ mice [40–43]. This may be the one of the main reasons for testicular atrophy and obstructed azoospermia in *Slc9a3*^*-/-*^ mice.

### Functional roles of SLC9A3 in the male reproductive tract

Mammalian spermatozoa are developed in the testis and undergo concentration and maturation in the seminiferous tubules, efferent ducts, epididymis, and vas deferens [38]. Precise regulation of pH and ion homeostasis is critical for luminal milieu [47]. SLC9A1, SLC9A2, SLC9A3, and SLC9A5, the Na^+^/H^+^ exchangers, are critical motional proteins in these processes [44,48]. SLC9A1–SLC9A3 are expressed in the efferent ducts and epididymis, and SLC9A1 and SLC9A5 are expressed in mature spermatozoa [44]. Loss of only *Slc9a1* or *Slc9a3* in male mice induces infertility. *Slc9a1*-null mice show severely decreased sperm motility because of disturbed intracellular pH in sperms [49]. SLC9A3 cooperates with ion channels such as CFTR and NBC1 and regulates H^+^ secretion and HCO_3_^-^ reabsorption in WT mice [50]. We found that *Slc9a3*^*-/-*^ mice displayed obstructed azoospermia-like phenotypes, which may be partially contributed to by decreased CFTR expression, similar to that observed in knockout (*cf/cf*) *Cftr* mice [39]. In addition, the dilated efferent ducts of *Slc9a3*^*-/-*^ mice were abnormally calcified. We suggest that aberrant secretion and calcification are likely due to the dysregulation of ion homeostasis and improper pH caused by SLC9A3 deficiency.

### Interdependent effects of CFTR and SLC9A3 in vivo

Ahn et al. indicated that SLC9A3 colocalized and interacted with CFTR in PS120 cells and mice pancreatic ducts [25]. In that study, the reduced SLC9A3 levels (53%) and activity in the pancreatic ducts of *△F/△F Cftr* mice were evaluated. The authors proposed that CFTR forms a complex with SLC9A3 and EBP53 to increase the stability of SLC9A3. In the present study, the markedly reduced CFTR expression in the epididymis and vas deferens caused by SLC9A3 deficiency was more deteriorated (85.7%–95.2%, Fig 1). This is consistent with and even more severe than that reported in the previous study [25]. We suggest that the obstructed azoospermia-like phenotypes in *Slc9a3*^-/-^ mice were attributable to both SLC9A3 deficiency and reduced CFTR expression.

### Comparison of the phenotypes of the male reproductive tract in *Slc9a3*^-/-^ and *Cftr*-null mice

The homozygous △F508 mutation (*△F/△F*) and knockout (*cf/cf*) *Cftr* mice, which show reduced male fertility, are two major strains of genetically modified mice with *Cftr* mutations [39,51]. In these strains, the development and morphology of the epididymis and vas deferens are normal before 20 days of age. At 40–44 days, spermatozoa are present in the testes and epididymis of *△F/△F* and *cf/cf* mice, but the lumen of the vas deferens contains abnormal secretions instead of spermatozoa and is narrower. These mouse strains are less fertile than the control mice, with only one in three *cf/cf* male mice being fertile. In the present study, we found that *Slc9a3*^-/-^ mice displayed similar but worse phenotypes including impaired spermatogenesis and less spermatozoa and severe aberrant secretions in the epididymis. Moreover, these results are consistent with the deteriorated levels of CFTR in *Slc9a3*^-/-^ mice reported in a previous study (Fig 1) [25].

### *Slc9a3* is a candidate gene of pathogenesis of CBAVD in Asian and Taiwanese populations

Large-scale genetic studies have indicated that genetic variants of *SLC9A3* are associated with early infection, lung infection severity, and susceptibility to meconium ileus in patients with CF [28–31]. However, the association between CBAVD, a mild phenotype of CF, and SLC9A3 is unclear. Recently, we detected the loss of a *SLC9A3* copy in Taiwanese patients with CBAVD by performing CGH and real-time PCR [17]. In the present study, we determined the detailed reproductive phenotypes, which are similar to the defects of *cf/cf* mice, in *Slc9a3*^-/-^ mice. These findings indicate a direct association between SLC9A3 and CBAVD in vivo. Because the phenotypes of deficiency or different mutations of *CFTR* in mouse models are quite distinct from those in swine models or humans [14,15,39,52], the pathogenesis in male excurrent ducts caused by SLC9A3 loss in different species should also be dissimilar. The association between SLC9A3 function and CBAVD must be clarified by collecting more CBAVD patients and performing clinical examinations of the epididymis and vas deferens in future.

## Materials and methods

### Ethics statement

The animal use protocol were reviewed and approved by the Institutional Animal Care and Use Committee (IACUC) of Fu Jen Catholic University (Approval number: A10064). The animal experiments were performed according to the international guidelines and regulations.

### Animals and fertile ability test

FVB.129(Cg)-*Slc9a3*^*tm1Ges*^/J mice were purchased from Jackson Laboratory. The genotype of each male mouse was assayed by extracting genomic DNA from the tail and by performing PCR. The primers used in genotyping were as follows: F1 (5ʹ-CATACAACATAGGACTAGCC-3'), R1 (5ʹ-CACTACTAGTCAGGCACTCT-3'), and R2 (5ʹ-CACTACTAGTCAGGCACTCT-3'). The primer ratio of F1, R1, and R2 was 2:1:1. More than 10 mice of each genotype were sacrificed at 2 months of age by anesthesia with isoflurane, and their organs, including the testes, epididymis, and vas deferens, were collected and weighed. The fertility and fecundity of WT and *Slc9a3*^*-/-*^ mice were compared by placing 2-month-old males of each genotype with two WT female mice and by counting the number of pups from each pregnancy.

### Immuno-blotting

Briefly, the organ tissues were homogenized in a lysis buffer [20 mM Tris/HCl (pH 8), 150 mM NaCl, 5 mM MgCl_2_, 0.5% Triton-X 100, 10% glycerol, and a protease inhibitor cocktail] and total protein extractions were heated for 5 min at 37°C before SDS-PAGE [53]. Antibodies against CFTR (ab2784; dilution, 1:3,000; Abcam, Cambridge, MA, USA) and actin (A5441; dilution, 1:20,000; Sigma-Aldrich, St Louis, MO, USA) were used and detected by chemiluminescence. Density was quantified using Image J software (National Institutes of Health, Bethesda, USA).

### Histological analysis and immunofluorescence staining

WT and *Slc9a3*^*-/-*^ mice were sacrificed at 2, 4, and 6 months of age, and their organs were collected. The testes were fixed in Bouin’s solution (Sigma-Aldrich), and the epididymis and vas deferens were fixed in PBS containing 4% paraformaldehyde. Next, the tissues were processed for embedding in paraffin wax. Sections of these paraffin-embedded tissues were stained with hematoxylin and eosin (H&E) for histological analysis. For immunofluorescence staining, the dewaxed sections were boiled with 0.1 M sodium citrate buffer (pH 6.0) for antigen retrieval. Sections were incubated overnight at 4°C with diluted primary antibodies including anti-SLC9A3 antibody (ab95299; Abcam, Cambridge, MA, USA) and anti-pan-keratin (4545; Cell Signaling, Beverly, MA, USA). Primary antibodies were detected with Alexa Fluor 488 and Alexa Fluor 568 fluorescent secondary antibodies (Invitrogen, Carlsbad, CA, USA) and followed by DAPI staining and mounted with Dako mounting medium. The acrosome was stained with lectin peanut agglutinin (L-32458; Invitrogen, Carlsbad, CA, USA). In all the experiments, at least three age-matched WT and *Slc9a3*^*-/-*^ mice were analyzed.

### Electron microscopy

Parts of the caput and cauda epididymis and vas deferens from 2-month-old mice were excised and immediately fixed with 4% paraformaldehyde and 0.1% glutaraldehyde overnight at 4°C. Next, the tissues were rinsed with 0.1 M phosphate buffer (pH 7.2) and treated with 1% osmium tetroxide at room temperature for 2 hours. After being rinsed with phosphate buffer again, the tissues were gradually dehydrated by series of increasing concentrations of ethanol. The tissues were then embedded with Spurr’s resin kit (cat-14300; EMS) overnight at room temperature. The embedded tissues were sectioned into 75-nm-thick sections by using an ultramicrotome (EM UC7, Leica Microsystems, Wetzlar, Germany) and mounted on copper grids. Ultramicrographs were acquired using a transmission electron microscope (JEM-1400; JEOL) at 100 Kva.

### Statistical analysis

All results were obtained from experiments performed in triplicate (at least) and are presented as the mean ± SEM. Data were analyzed with a Student’s *t* test to determine the significance between two groups. Differences with a p value of *<*0.05 were considered statistically significant.

## Supporting information

S1 Fig Specificity of anti-SLC9A3 antibody analyzed in the present study. (A, B) Immunofluorescence double staining was performed with anti-SLC9A3 antibody (green) and anti-pan-cytokeratin antibody (red) on the caput (A) and cauda (B) of two *Slc9a3*-deficient mice. The nucleus was stained with DAPI (blue). There was no green signal on the *Slc9a3*^*-/-*^ epididymal sections. (C-D) Specificity of the anti-SLC9A3 antibody was confirmed on the liver, spleen, heart (C), and smooth muscle (D). These tissue sections were used as negative controls for the specificity of anti-SLC9A3 antibody. According to the EST profile Mm.261564 on UniGene, SLC9A3 is not expressed in these mouse tissues. (C) SLC9A3 signaling was not detected in the liver, spleen, or heart. The lower panel is the H&E staining sections showing the morphology of the tissues we used. (D) Section showing the blood vessel composed of endothelial and smooth muscle cells. Alpha smooth muscle actin (red) was used as a specific marker of smooth muscle cells. The nonspecific binding of anti-SLC9A3 antibody was not detected on the smooth muscle. Scale bar = 20 μm (A-D) or 200 μm (C, lower panel).
(TIF)

S2 Fig Number of elongating spermatids at stage VI–VII of spermatogenesis are reduced in *Slc9a3*^*-/-*^ mice. Testicular sections of 6-month-old WT (A, B) and *Slc9a3*^*-/-*^ mice of different ages (C–H) were stained with lectin peanut agglutinin (red), which was used as a specific marker of acrosome. Each acrosome (red) represents an elongating spermatid. The nucleus was stained with DAPI (blue) and is displayed in merged images. The insets represent higher magnification of the boxed areas; scale bar = 20 μm. (I) Quantification of the number of elongating spermatids on the testicular sections of WT and *Slc9a3*^*-/-*^ mice. The elongating spermatids, a type of germ cell, at stage VI–VII during spermatogenesis were selected to reflect the size of the entire population of germ cells. The number of elongating spermatids in a seminiferous tubule was counted, and 10 seminiferous tubules of each mouse were scored. The data are presented as the acrosome number per area (1 × 10^4^ μm^2^). Three WT and three *Slc9a3*^*-/-*^ mice of different ages were analyzed. Each bar is the mean ± SEM; *n* = 3 per group. *Significant difference compared with WT mice (***p < 0.0001, analyzed using the Student’s *t* test).
(TIF)

S3 Fig Absence of spermatozoa in the caput epididymis and vas deferens of 2-month-old *Slc9a3*^*-/-*^ mice. Epididymal sections of WT (A, C) and *Slc9a3*^*-/-*^ mice (B, D) were deparaffinized and rehydrated. The nucleus was stained with DAPI (blue) and represents the numbers of cells and spermatozoa. (A, B) General view of the caput epididymis. (C, D) Enlarged images showing the region selected in the low-magnification image. Arrows indicate the spermatozoa; scale bar = 20 μm. (E, F) Mature spermatozoa were flushed from the vas deferens of WT mice (E) but not from the vas deferens of *Slc9a3*^*-/-*^ mice (F) by using an HTF medium. The inset shows higher magnification of the indicated areas. (G) The percentage of lumens with spermatozoa was quantified. The presence of spermatozoa was determined in 20 lumens of each caput epididymis sample. Each bar is the mean ± SEM; *n* = 6 per genotype. *Significant difference compared with WT mice (***p < 0.0001, analyzed using an unpaired Student’s *t* test).
(TIF)

S4 Fig Higher magnification image of smooth endoplasmic reticulum-like material within particles in the lumen of the vas deferens of *Slc9a3*^*-/-*^ male mice. The secretory vesicles are surrounded by some stereocilia. Smooth endoplasmic reticulum in the epithelial cells of the vas deferens displays whorls-like structures or flattened saccules organized in a parallel array in the vas deferens epithelium; scale bar = 2 μm.
(TIF)
