# Supplementary figures and images for: Loss of SLC9A3 decreases CFTR protein and causes obstructed azoospermia in mice
Source: PLoS Genet. 2017 Apr 6;13(4):e1006715. doi: 10.1371/journal.pgen.1006715 (PMC5398719; doi:10.1371/journal.pgen.1006715)

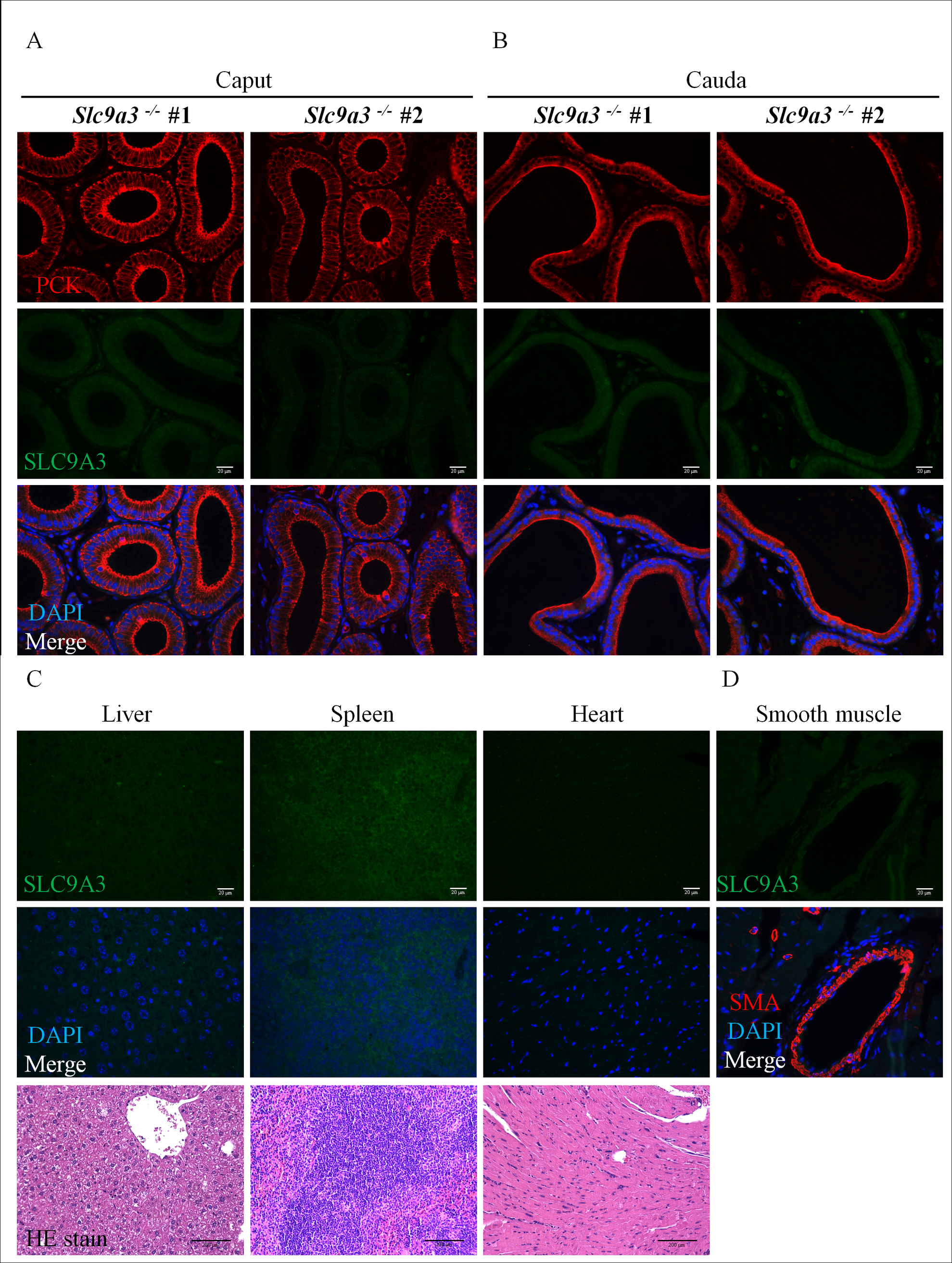

Supplement: S1 Fig — (A, B) Immunofluorescence double staining was performed with anti-SLC9A3 antibody (green) and anti-pan-cytokeratin antibody (red) on the caput (A) and cauda (B) of two Slc9a3-deficient mice. The nucleus was stained with DAPI (blue). There was no green signal on the Slc9a3-/- epididymal sections. (C-D) Specificity of the anti-SLC9A3 antibody was confirmed on the liver, spleen, heart (C), and smooth muscle (D). These tissue sections were used as negative controls for the specificity of anti-SLC9A3 antibody. According to the EST profile Mm.261564 on UniGene, SLC9A3 is not expressed in these mouse tissues. (C) SLC9A3 signaling was not detected in the liver, spleen, or heart. The lower panel is the H&E staining sections showing the morphology of the tissues we used. (D) Section showing the blood vessel composed of endothelial and smooth muscle cells. Alpha smooth muscle actin (red) was used as a specific marker of smooth muscle cells. The nonspecific binding of anti-SLC9A3 antibody was not detected on the smooth muscle. Scale bar = 20 μm (A-D) or 200 μm (C, lower panel). (TIF) [file pgen.1006715.s001.tif]

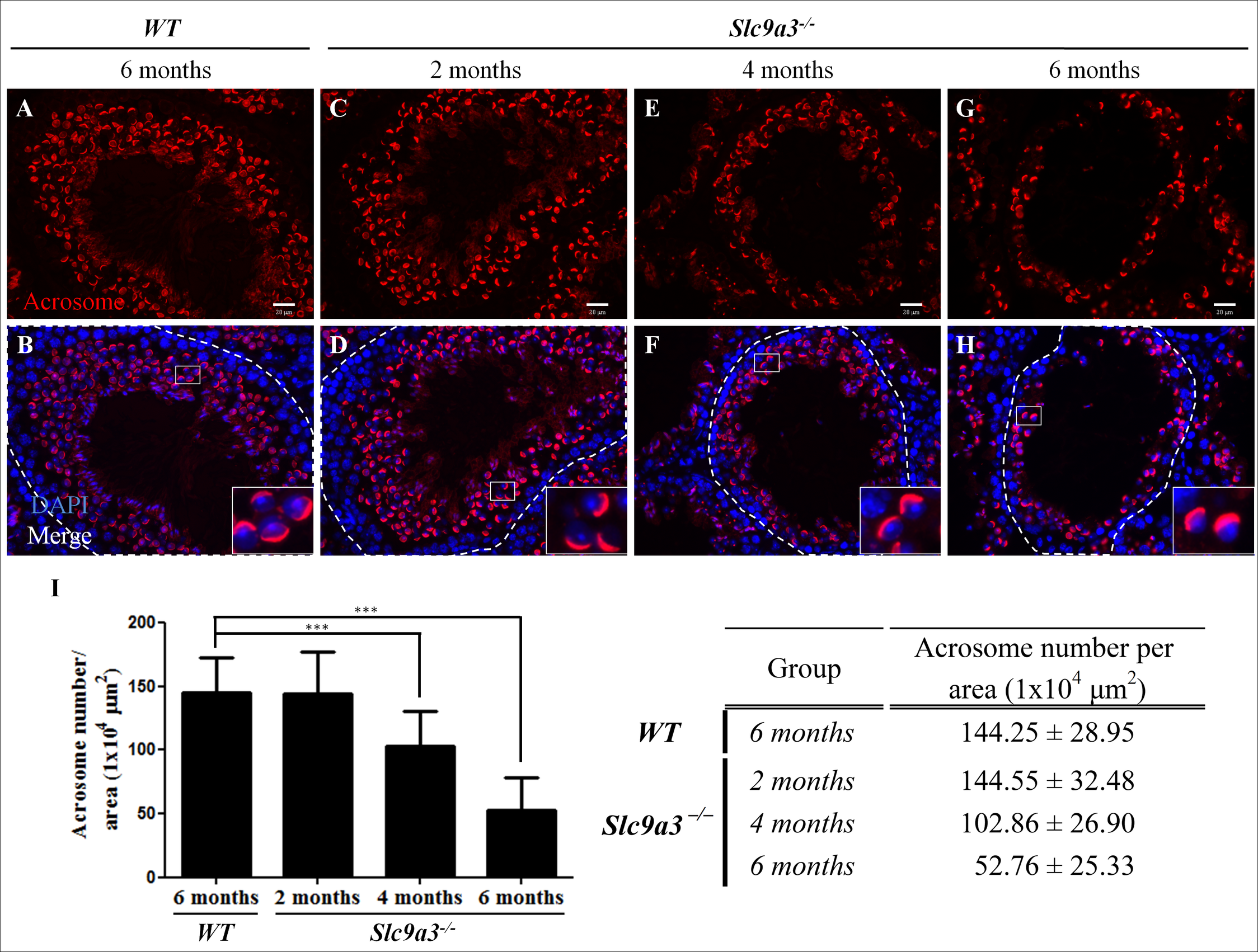

Supplement: S2 Fig — Testicular sections of 6-month-old WT (A, B) and Slc9a3-/- mice of different ages (C–H) were stained with lectin peanut agglutinin (red), which was used as a specific marker of acrosome. Each acrosome (red) represents an elongating spermatid. The nucleus was stained with DAPI (blue) and is displayed in merged images. The insets represent higher magnification of the boxed areas; scale bar = 20 μm. (I) Quantification of the number of elongating spermatids on the testicular sections of WT and Slc9a3-/- mice. The elongating spermatids, a type of germ cell, at stage VI–VII during spermatogenesis were selected to reflect the size of the entire population of germ cells. The number of elongating spermatids in a seminiferous tubule was counted, and 10 seminiferous tubules of each mouse were scored. The data are presented as the acrosome number per area (1 × 10^4^ μm2). Three WT and three Slc9a3-/- mice of different ages were analyzed. Each bar is the mean ± SEM; n = 3 per group. *Significant difference compared with WT mice (***p < 0.0001, analyzed using the Student’s t test). (TIF) [file pgen.1006715.s002.tif]

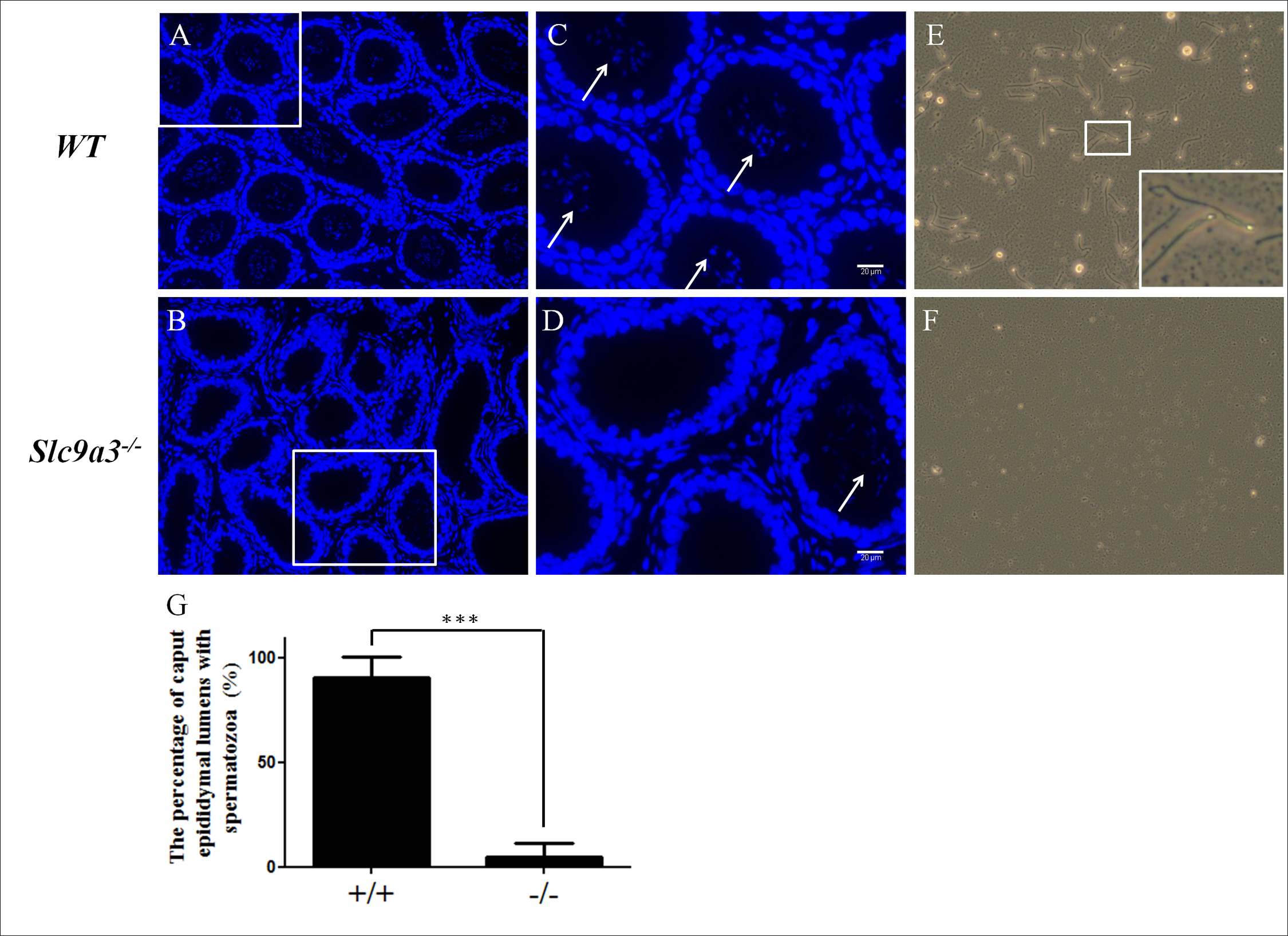

Supplement: S3 Fig — Epididymal sections of WT (A, C) and Slc9a3-/- mice (B, D) were deparaffinized and rehydrated. The nucleus was stained with DAPI (blue) and represents the numbers of cells and spermatozoa. (A, B) General view of the caput epididymis. (C, D) Enlarged images showing the region selected in the low-magnification image. Arrows indicate the spermatozoa; scale bar = 20 μm. (E, F) Mature spermatozoa were flushed from the vas deferens of WT mice (E) but not from the vas deferens of Slc9a3-/- mice (F) by using an HTF medium. The inset shows higher magnification of the indicated areas. (G) The percentage of lumens with spermatozoa was quantified. The presence of spermatozoa was determined in 20 lumens of each caput epididymis sample. Each bar is the mean ± SEM; n = 6 per genotype. *Significant difference compared with WT mice (***p < 0.0001, analyzed using an unpaired Student’s t test). (TIF) [file pgen.1006715.s003.tif]

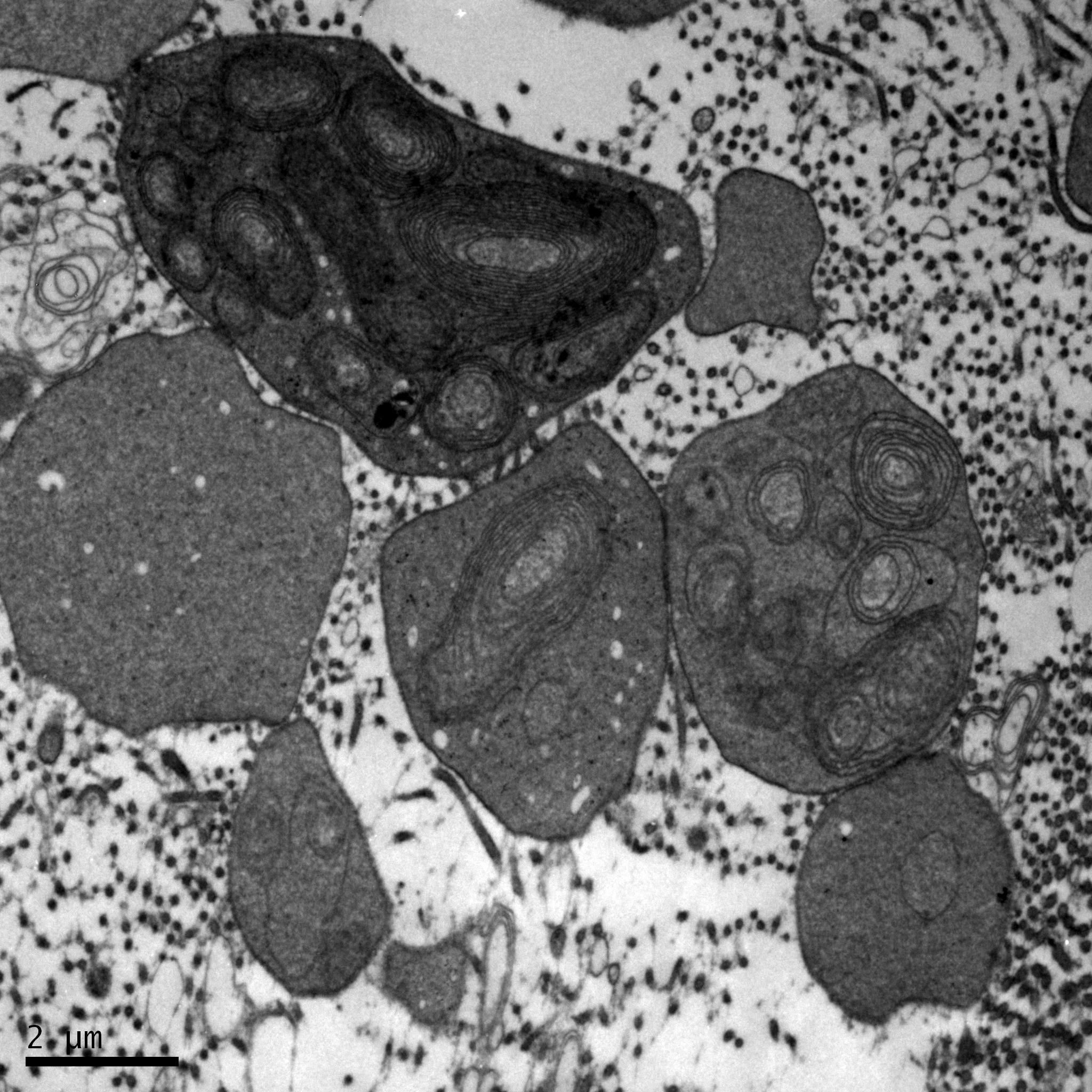

Supplement: S4 Fig — The secretory vesicles are surrounded by some stereocilia. Smooth endoplasmic reticulum in the epithelial cells of the vas deferens displays whorls-like structures or flattened saccules organized in a parallel array in the vas deferens epithelium; scale bar = 2 μm. (TIF) [file pgen.1006715.s004.tif]
